# A counterpoint paper: Comments on the electrocardiographic part of the 2018 Fourth Universal Definition of Myocardial Infarction endorsed by the International Society of Electrocardiology and the International Society for Holter and Noninvasive Electrocardiology

**DOI:** 10.1111/anec.12786

**Published:** 2020-07-08

**Authors:** Yochai Birnbaum, Miguel Fiol, Kjell Nikus, Javier Garcia Niebla, Ljuba Bacharova, Sergio Dubner, Wojciech Zareba, Peter W. Macfarlane, Antonio Luiz Ribeiro, Iwona Cygankiewicz, Antoni Bayes de Luna

**Affiliations:** ^1^ The Department of Medicine The Section of Cardiology Baylor College of Medicine Houston TX USA; ^2^ Health Research Institute of the Balearic Islands Hospital Son Espases Palma Spain; ^3^ Faculty of Medicine and Health Technology Finnish Cardiovascular Research Center University of Tampere Tampere Finland; ^4^ Heart Hospital Tampere University Hospital Tampere Finland; ^5^ Servicios Sanitarios del Area de Salud de El Heirro Valle del Golfo Health Center Frontera Spain; ^6^ International Laser Center Bratislava Slovak Republic; ^7^ Institute of Pathophysiology Medical School Comenius University Bratislava Slovak Republic; ^8^ Clinica y Maternidad Suizo Argentina and De Los Arcos Sanatorio Buenos Aires Argentina; ^9^ Division of Cardiology University of Rochester Medical Center Rochester NY USA; ^10^ Electrocardiology Section Institute of Health and Wellbeing University of Glasgow Glasgow UK; ^11^ Internal Medicine Department School of Medicine, and Telehealth Center Hospital das Clínicas Universidade Federal de Minas Gerais Belo Horizonte Brazil; ^12^ Department of Electrocardiology Medical University of Lodz Lodz Poland; ^13^ Cardiovascular ICCC‐ Program Research Institute Hospital de la Santa Creu i Sant Pau Cardiovascular Research Foundation Barcelona Spain

**Keywords:** electrocardiography, epidemiology/clinical trials, non‐invasive techniques

## Abstract

The Fourth Universal Definition of Myocardial Infarction (FUDMI) focuses on the distinction between nonischemic myocardial injury and myocardial infarction (MI), along with the role of cardiovascular magnetic resonance, in order to define the etiology of myocardial injury. As a consequence, there is less emphasis on updating the parts of the definition concerning the electrocardiographic (ECG) changes related to MI. Evidence of myocardial ischemia is a prerequisite for the diagnosis of MI, and the ECG is the main available tool for (a) detecting acute ischemia, (b) triage, and (c) risk stratification upon presentation. This review focuses on multiple aspects of ECG interpretation that we firmly believe should be considered for incorporation in any future update to the Universal Definition of MI.

## 1 INTRODUCTION

The Fourth Universal Definition of Myocardial Infarction (FUDMI), published simultaneously in 2018 in numerous journals including Circulation, Journal of the American College of Cardiology and European Heart Journal, focuses mainly on the distinction between nonischemic myocardial injury and myocardial infarction (MI) and the role of cardiovascular magnetic resonance in defining the etiology of myocardial injury, with less emphasis on updating the parts related to the electrocardiographic (ECG) changes related to MI (Thygesen et al., 2019).

Evidence of myocardial ischemia is a prerequisite for the diagnosis of MI and the ECG is the main diagnostic tool for detecting acute myocardial ischemia, as stated in the document, viz “Myocardial ischemia in a clinical setting can most often be identified from the patient's history and from the ECG” (Thygesen et al., 2019). Therefore, the definitions of “ischemic changes” should be accurate. The International Society of Electrocardiology (ISE) and the International Society for Holter and Noninvasive Electrocardiology (ISHNE) focus on the ECG and have members that are expert in interpreting ECG changes detected during ischemia and infarction. This counterpoint review focuses on several topics related to the ECG that we believe should be considered to be modified and incorporated into future versions of the document.

## 2 METHODS

YB, MF, KN, JGN, and AB read the FUDMI and communicated about topics related to the ECG that should be discussed in this article. Those ideas were incorporated into an initial draft by YB that has been circulated to all co‐authors for review and approval. Communications were by email, Skype and in person during The International Society of Electrocardiology (ISE) and the International Society for Holter and Noninvasive Electrocardiology (ISHNE) conference in Belgrade 2019. The manuscript has been updated accordingly, until all co‐authors approved the final version.

The headings in this article refer mainly to the original sections of the FUDMI (Thygesen et al., 2019).

## 3 MYOCARDIAL INFARCTION ASSOCIATED WITH CORONARY ARTERY BYPASS GRAFTING (TYPE 5 MYOCARDIAL INFARCTION)

Although the FUDMI states “It is important that the postprocedural elevation of cTn values is accompanied by ECG, angiographic, or imaging evidence of new myocardial ischemia/new loss of myocardial viability,” the thresholds for the ECG changes, especially ST deviation, are not mentioned. The document specifies that ST‐T changes are common after coronary artery bypass grafting (CABG) due to epicardial injury and are not reliable indicators of myocardial ischemia in this setting. “However, ST elevation with reciprocal ST depression or other specific ECG patterns may be a more reliable finding of a potential ischemic event” (Thygesen et al., 2019).

1. While later on, ST‐T changes can be secondary to pericardial/epicardial inflammation and, thus, can be nonspecific (Borgaonkar & Birnbaum, 2019), soon after completion of cardiac surgery they are probably more specific (Liu & Birnbaum, 2019). At this stage, when the patient is still sedated and intubated, ST deviation can be an early marker of acute bypass failure or type 5 MI. Further studies are needed to evaluate the accuracy of routine 12‐lead ECG after completion of surgery to detect ischemia/ infarction.
2. As the thresholds for cardiac troponin elevation for diagnosing MI are different for type 5 MI (>10 times the 99th percentile of the upper limit of normal) than for the other types of MI, it might be that different thresholds of ST deviation should be used in this scenario. As mentioned above, further studies are needed to clarify this issue.
3. Reciprocal changes are commonly seen in STEMI with ST elevation in the limb leads (inferior or lateral STEMI) (Birnbaum, Sclarovsky, Mager, Strasberg, & Rechavia, 1993). However, they are less common in anterior STEMI, especially when the left anterior descending (LAD) is occluded after the first diagonal branch (Birnbaum, Nikus, et al., 2014). In our experience, acute MI caused by anastomosis failure or distal embolization of a graft to the LAD (usually the insertion is distal) normally does not cause reciprocal ST depression.

## 4 ELECTROCARDIOGRAPHIC DETECTION OF MYOCARDIAL INFARCTION

As stated, “The ECG is an integral part of the diagnostic workup of patients with suspected MI, and should be acquired and interpreted promptly,” we disagree with the statement that “more profound ST‐segment shifts or T wave inversion involving multiple leads/ territories are associated with a greater degree of myocardial ischemia…”. In our experience and based on the literature, T wave inversion in leads with an isoelectric ST segment does not occur with acute ischemia. It can be seen in the subacute phase or after reperfusion and should be regarded as “postischemic changes” (de Luna et al., 2014). Negative T waves in leads with ST elevation are also seen after reperfusion or in the subacute phase of infarction. Only when seen in leads with ST depression can negative T waves signify acute subendocardial ischemia, or changes reciprocal to ST elevation in opposing leads. Therefore, we believe that T wave inversion should not be considered a sign of active ischemia.

It is written that “ST‐segment depression ≥ 1 mm in 6 leads, which may be associated with ST segment elevation in leads aVR or lead V_1_ and hemodynamic compromise, is suggestive evidence of multivessel disease or left main disease.” However, in our opinion this statement should probably be restricted to patients in the appropriate clinical situation and to those with a relatively normal baseline ECG (Kim & Birnbaum, 2013; Knotts, Wilson, Kim, Huang, & Birnbaum, 2013). In many patients with left ventricular hypertrophy, critical aortic stenosis, cardiomyopathy, left bundle branch block, or nonspecific intraventricular conduction delay, dynamic diffuse ST depression associated with ST elevation in aVR can be seen. These changes can be more pronounced in patients with tachycardia or increased afterload. Implementation of the original statement could lead to overdiagnosing NSTEMI in patients with positive cardiac markers secondary to hypertensive crisis or exacerbation of acute heart failure.

**FIGURE 1:**
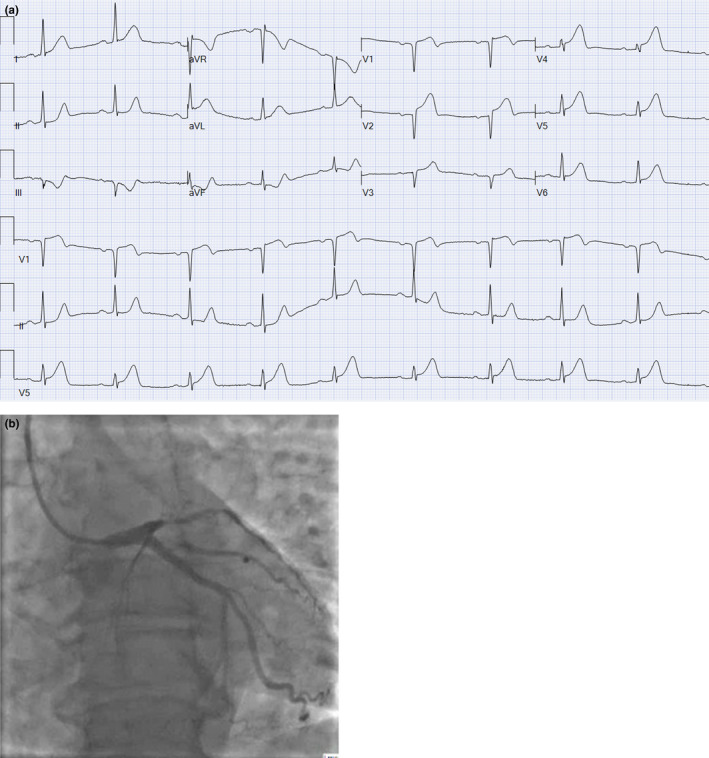
(a) A presenting ECG of a patient with anterior STEMI. There is concave ST elevation in V_1_–V_6_. There is a reciprocal ST depression in III and aVF. (b) Emergent coronary angiography shows tight lesion in the proximal left anterior descending (LAD) coronary artery

We think that the next statement is also questionable: “Prolonged new convex ST‐segment elevation, particularly when associated with reciprocal ST‐segment depression, usually reflects acute coronary occlusion and results in myocardial injury with necrosis.” The traditional literature emphasizes the “convex” pattern. Probably in the pre‐reperfusion era when patients presented late (often with T wave inversion in the leads with ST elevation), the ST was often convex. However, nowadays, when patients present early, in a large percentage of patients, the ST is concave (Huang & Birnbaum, 2011). This is especially common in patients with anterior STEMI presenting early with ST elevation and tall positive T waves (Figure 1).

**FIGURE 2:**
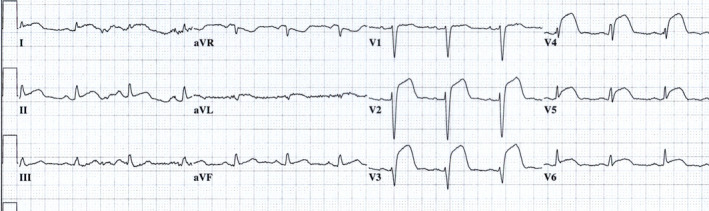
Anterior STEMI with ST elevation in I, aVL, V_2_–V_6_ without reciprocal ST depression

As mentioned above, reciprocal changes are commonly seen in inferior or lateral STEMI with ST elevation in the limb leads. However, they are less common in anterior infarct, especially when the LAD is occluded after the first diagonal branch. Yet, occlusion of a short LAD before the first diagonal branch is usually associated with ST elevation in aVL and reciprocal ST depression in the inferior leads. However, in the majority of patients with anterior STEMI, reciprocal ST depression is not seen (Figure 2).

“Reciprocal changes can help to differentiate STEMI from pericarditis or early repolarization changes.” Indeed, in both early repolarization and acute pericarditis, reciprocal changes are commonly seen only in aVR. Yet, in patients with left ventricular hypertrophy, cardiomyopathy, and/or LBBB, “reciprocal” changes are common (ST elevation in V_1_–V_2_ with ST depression in I, aVL, V_5_–V_6_) (Birnbaum & Alam, 2014).

We believe that the cutoffs of ST‐segment elevation for the different leads (table 2 in the FUDMI) should probably be limited to patients with narrow QRS and without voltage criteria for LVH (Macfarlane et al., 2004). [I introduced some of this in JECG 2004;37(Suppl): 98–103 and did say at that time that the criteria did not apply in the presence of LVH.] The thresholds for ischemic ST elevation in patients with LVH or cardiomyopathies have not been established (Birnbaum & Alam, 2014) (Macfarlane et al., 2004).

Moreover, many patients with right bundle branch block (RBBB) display ST depression in leads V_1_–V_3_ at baseline. There are no established guidelines on how to diagnose acute inferolateral STEMI (ST depression in the anterior leads) in patients with complete or incomplete RBBB (Wei et al., 2013). In addition, it is unclear whether lower thresholds should be used for ST elevation in the anterior leads for diagnosing anterior STEMI in patients with complete or incomplete RBBB (Wei et al., 2013).

It should be remembered that due to the way in which the six limb leads are derived, “reciprocal” changes within these leads are merely a function of the lead derivation, for example, since aVR = −½(I + II), if there is ST depression in Leads I and II, then by definition, there must be ST elevation in avR. Simple mathematical considerations also show that ST elevation in aVL is reflected in ST depression in III and aVF so in a way these “reciprocal” changes in limb leads are “automatic” changes.

While the text states that “upsloping ST‐segment depression > 1 mm at the J‐point in the precordial leads” can be a sign of “significant left anterior descending artery (LAD) occlusion,” this pattern has not been included in table 2 of the FUDMI. Upsloping ST depression is commonly seen during exercise stress tests, and there is controversy regarding its significance. While initially it was considered a nonspecific pattern induced by tachycardia and not specific for ischemia, more recent studies have suggested that upsloping ST depression can be a true indicator of ischemia (Polizos & Ellestad, 2006; Rijneke, Ascoop, & Talmon, 1980) (de Winter et al., 2019; de Winter, Adams, Verouden, & de Winter, 2016). Upsloping ST depression with tall T waves in the anterior leads was described as an ECG sign of proximal LAD occlusion in patients presenting with chest pain (de Winter, Verouden, Wellens, & Wilde, 2008; Verouden et al., 2009). More recently, this pattern was described in 11 patients (0.2%) of 5,588 with suspected acute coronary syndromes whose ECG was transmitted by the field triage team. All of them had a culprit lesion in the proximal LAD (de Winter et al., 2019; de Winter et al., 2016). However, there are anecdotal descriptions of a similar pattern of upsloping ST depression with tall T waves in left circumflex ischemia (Alam, Nikus, Fiol, Bayes de Luna, & Birnbaum, 2019; Birnbaum, Wilson, et al., 2014; Misumida, Kobayashi, Schweitzer, & Kanei, 2015) and even right coronary artery ischemia (Tsutsumi & Tsukahara, 2018). Thus, we think that the description be changed to “significant coronary artery occlusion,” rather than LAD occlusion based on the current literature.

It should also be noted that normal limits of ST elevation are race‐dependent (Macfarlane et al., 2014). African and Chinese males, for example, have higher normal limits of ST elevation compared with Caucasians particularly in precordial leads. We think that allowance therefore has to be made for interpretation of ST shift particularly in Africans and Chinese.

## 5 APPLICATION OF SUPPLEMENTAL ELECTROCARDIOGRAM LEADS

The FUDMI recommends the use of “posterior leads at the fifth intercostal space (V_7_ at the left posterior axillary line, V_8_ at the left midscapular line, and V_9_ at the left paraspinal border)” for detecting ischemia caused by left circumflex occlusion. However, the original description of Wilson et al was that leads V_6_–V_8_ will be placed on a horizontal line from lead V_4_, rather than following the fifth intercostal space ("RECOMMENDATIONS for standardization of electrocardiographic and vectorcardiographic leads," Wilson et al., 1954) as used by Matetzky et al in the reference quoted by the FUDMI (Matetzky et al., 1999) and as described by the 2007 AHA/ACC/HRS Scientific Statement for the recommendations for the standardization and interpretation of the electrocardiogram (Kligfield et al., 2007).

There is specific recommendation for recording these leads “in patients with high clinical suspicion of acute circumflex occlusion (e.g., initial ECG nondiagnostic or ST‐segment depression in leads V_1_–V_3_)”. However, ST‐segment depression in leads V_1_–V_3_ is not suggestive of inferobasal myocardial ischemia. The RCA courses on the right atrioventricular groove, supplying branches to the right atrium and the free wall of the right ventricle, until the junction with the posterior interventricular groove. This can give a posterior descending artery that courses along the posterior interventricular groove and/or a posterolateral branch that supplies the inferolateral segments. The LCX courses on the left atrioventricular groove, until it reaches the posterior interventricular groove, supplying the left atrium and the free wall of the left ventricle via the obtuse marginal branches. The LCX may give rise to the left posterior descending artery that travels along the posterior interventricular groove. Thus, the majority of inferior infarcts due to RCA or LCX occlusion involve the basal inferior segment, unless the occlusion is in the mid or distal part of the posterior descending artery, sparing the basal segments. Cardiac MRI‐ECG correlation suggested that it is correlated with the projection of the vector of inferior ischemia on the anterior‐posterior plan (Jia et al., 2018).

In the acute phase of STEMI, the reciprocal changes of ST elevation with positive T waves are ST depression with negative T waves. Therefore, in the acute stages of inferolateral STEMI with ST depression in V_1_–V_3_, the T waves are usually negative. Only after reperfusion, or with a more advanced stage of infarction, do the T waves become positive (reciprocal changes of ST elevation with negative T waves) (Porter et al., 1998).

## 6 CONDUCTION DISTURBANCES AND PACEMAKERS

The FUDMI states “In patients with LBBB, ST‐segment elevation ≥ 1 mm concordant with the QRS complex in any lead may be an indicator of acute myocardial ischemia.” Yet, the sensitivity of this sign for STEMI physiology (acute occlusion of an epicardial artery) is low. We suggest that it be stated that since detection of ischemia by the ECG in LBBB is difficult, decisions concerning urgent reperfusion therapy should be based mainly on symptoms and hemodynamic parameters. According to the 2013 ACCF/AHA STEMI guidelines “New or presumed new LBBB has been considered a STEMI equivalent.” Most cases of LBBB at time of presentation, however, are “not known to be old” because a prior ECG is not available for comparison. New or presumed new LBBB at presentation occurs infrequently, may interfere with ST‐elevation analysis, and should not be considered diagnostic of acute MI in isolation” (Jain et al., 2011; O'Gara et al., 2013). The European guidelines, published in 2017, specify “In the presence of LBBB, the ECG diagnosis of acute myocardial infarction is difficult but often possible if marked ST‐segment abnormalities are present. Somewhat complex algorithms have been offered to assist the diagnosis, but they do not provide diagnostic certainty. The presence of concordant ST‐segment elevation (i.e. in leads with positive QRS deflections) appears to be one of the best indicators of ongoing MI with an occluded infarct artery. On the other hand, ST depression (usually concordant) in V_1_–V_3_ in LBBB is very specific but not overly sensitive (Sgarbossa et al., 1996). Patients with a clinical suspicion of ongoing myocardial ischemia and LBBB should be managed in a way similar to STEMI patients, regardless of whether the LBBB is previously known. It is important to remark that the presence of a (presumed) new LBBB does not predict an MI per se” (Ibanez et al., 2018).

The FUDMI states “New, or presumed new, RBBB without associated ST‐segment or T wave changes is associated with thrombolysis in myocardial infarction (TIMI) 0–2 flow in as many as 66% of patients (compared with > 90% in those with ST‐segment or T wave changes).” The 2017 ESC guidelines for STEMI also specify “Patients with myocardial infarction and RBBB have a poor prognosis. It may be difficult to detect transmural ischemia in patients with chest pain and RBBB. Therefore, a primary percutaneous coronary intervention strategy (emergent coronary angiography and percutaneous coronary intervention if indicated) should be considered when persistent ischemic symptoms occur in the presence of RBBB” (Ibanez et al., 2018). These recommendations are mainly based on a retrospective study by Widimsky et al. (2012). These authors analyzed 6,742 patients with acute MI and found that among the 427 patients with RBBB (53% with concomitant ST elevation), TIMI flow 0 in the infarct‐related artery was present in 51.7% and primary percutaneous coronary intervention was performed in 80.1% of the patients. TIMI flow 0 in the infarct‐related artery was found in significantly more patients with new or presumed new RBBB (55%) than in the group with old RBBB 34.9%, old LBBB 28% or new or presumed new LBBB 41.1%. We think it should be stressed that these results apply to patients with adjudicated acute MI, rather than for the general population of patients presenting with acute symptoms. Moreover, the patients included in the study underwent cardiac catheterization, not necessarily emergent catheterization, as a part of a primary percutaneous coronary intervention protocol. Significant coronary artery lesions, including chronic total occlusion, can be found in patients with stable coronary artery disease and in those with non‐ST‐elevation acute coronary syndromes. Thus, coronary angiography can be recommended. However, as there are no data on outcomes with and without primary percutaneous coronary intervention in patients with chest pain and presumed new RBBB without ST deviation, the recommendation for emergent coronary angiography is probably overreaching. Recommending primary percutaneous coronary intervention for patients presenting with RBBB and with atypical symptoms (shortness of breath, acute heart failure, etc.) should be prospectively tested. A more recent study by Neumann et al. retrospectively assessed the significance of RBBB in patients presenting with suspected myocardial infarction. Among 4,067 patients presenting with compatible symptoms, 125 patients (3.1%) had RBBB. Only 23 of them (18.4%) had a final diagnosis of acute MI (6 had STEMI and 17 NSTEMI). Yet, one‐year mortality for patients with RBBB was 10.7%. They concluded that their data support the new ESC statement that RBBB is associated with a high risk of mortality; however, as the likelihood of acute MI was comparable to that of the patients without RBBB, they questioned the indication for emergent coronary angiography solely based on the presence of RBBB (Neumann et al., 2019). In our experience, diagnosing ST elevation in the inferior and lateral leads can easily be done in patients with RBBB. The problem, as discussed earlier, is how to diagnose inferolateral STEMI equivalent in patients with RBBB and baseline ST depression in V_1_–V_3_ and whether the threshold for ST elevation in the anterior leads V_1_–V_3_ should be reduced.

## 7 ADDITIONAL COMMENTS

### 7.1 Reperfused STEMI

Although the FUDMI does not directly deal with indications for acute reperfusion therapy, the entity of (spontaneously) reperfused STEMI is not mentioned in the documents nor in the STEMI guidelines (Ibanez et al., 2018; O'Gara et al., 2013). Patients with spontaneous reperfusion at presentation (improvement in symptoms and ST‐elevation resolution compared with a previous ECG) have a good prognosis without primary percutaneous coronary intervention (de Luna et al., 2014; Dowdy et al., 2004). As they do not have ongoing ischemia with progression of necrosis, coronary revascularization is indicated to prevent re‐ischemia/ re‐infarction, rather than salvaging myocardium. This can be done urgently, as in high‐risk NSTEMI, rather than by using the time frame of primary percutaneous coronary intervention for acute STEMI. The most recent ESC STEMI guidelines from 2017 state “Early angiography (within 24 hr) is recommended if symptoms are completely relieved and ST‐segment elevation is completely normalized spontaneously or after nitroglycerin administration (provided there is no recurrence of symptoms or ST‐segment elevation).” Yet, those with resolution of symptoms and persistent ST elevation (despite having clear decrease in the magnitude of ST elevation compared to the original ECG) should be treated according to this indication: “Reperfusion therapy is indicated in all patients with symptoms of ischemia of ≤ 12 hr duration and persistent ST‐segment elevation” (Ibanez et al., 2018; O'Gara et al., 2013; Steg et al., 2012). This statement does not emphasize “ongoing” symptoms and therefore includes patients in whom symptoms resolved but who continued to have ST elevation above the relevant threshold. We believe that thrombolytic therapy should not be administered to patients with spontaneous reperfusion despite having residual ST elevation, as the current recommendation states: “If timely primary PCI cannot be performed after STEMI diagnosis, fibrinolytic therapy is recommended within 12 hr of symptom onset in patients without contraindications” (Ibanez et al., 2018; O'Gara et al., 2013; Steg et al., 2012).

## 8 CONCLUSION

In conclusion, although the ECG has been used for the diagnosis and triage of patients with suspected myocardial infarction for many decades, many of the concepts and terminology currently used should be updated, as in other fields of cardiology and imaging. As new information is available, based on high‐sensitive biomarkers, angiography, and especially cardiac magnetic resonance images, our understanding of the significance of the various ECG patterns continues to be refined. We recommend that these new concepts should be further studied and given due consideration for incorporation in any future guidelines.

## CONFLICT OF INTEREST

The authors declare that they have no conflict of interest.

## AUTHOR CONTRIBUTIONS

All authors participate in writing and editing the manuscript.
